# Crystal structure of poly[[μ-4-(hy­droxy­meth­yl)pyridine-κ^2^ *N*:*O*][4-(hy­droxy­meth­yl)pyridine-κ*N*](μ-thio­cyanato-κ^2^ *N*:*S*)(thio­cyanato-κ*N*)cadmium]

**DOI:** 10.1107/S2056989015008890

**Published:** 2015-05-13

**Authors:** Julia Werner, Inke Jess, Christian Näther

**Affiliations:** a Institut für Anorganische Chemie, Christian-Albrechts-Universität Kiel, Max-Eyth-Strasse 2, 24118 Kiel, Germany

**Keywords:** crystal structure, coordination polymer, cadmium, octa­hedral coordination, hydrogen bonding

## Abstract

The crystal structure of the title compound, [Cd(NCS)_2_(C_6_H_7_NO)_2_]_*n*_ is made up of Cd^2+^ cations that are coordinated by three thio­cyanate ligands and three 4-(hy­droxy­meth­yl)pyridine ligands within distorted N_4_OS octa­hedra. The asymmetric unit consists of one Cd^2+^ cation, two thio­cyanate anions and two 4-(hy­droxy­meth­yl)pyridine ligands in general positions. Two Cd^2+^ cations are linked by two μ-1,3 *N*- and *S*-bonding thio­ycanate anions into dimers which are further linked into branched chains along [100] by two μ-1,6 *N*- and *O*-bonding 4-(hy­droxy­meth­yl)pyridine ligands. One additional *N*-bonded 4-(hy­droxy­meth­yl)pyridine ligand and one additional *N*-bonded thio­cyanate anion are only terminally bonded to the metal cation. Inter­chain O—H⋯S hydrogen bonds between the hy­droxy H atoms and one of the thio­cyanate S atoms connect the chains into a three-dimensional network.

## Related literature   

For similar structures with thio­cyanate anions in bridging coordination to cadmium, see: Banerjee *et al.* (2005 ▸); Tahli *et al.* (2011 ▸).
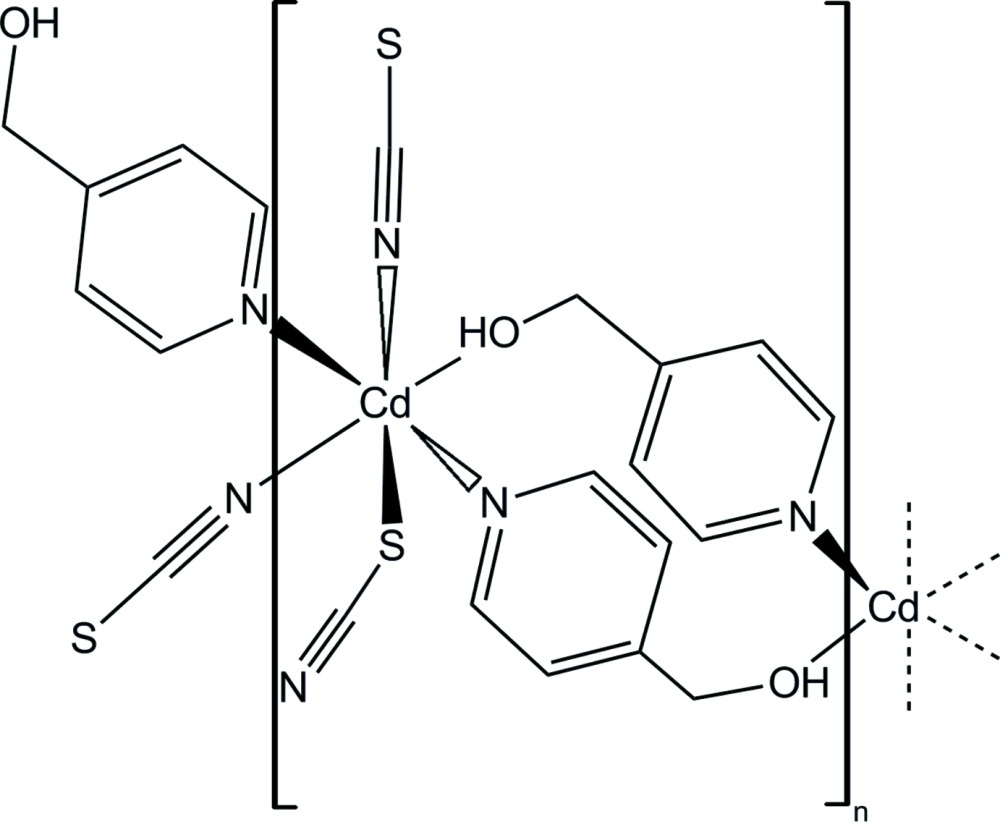


## Experimental   

### Crystal data  

[Cd(NCS)_2_(C_6_H_7_NO)_2_]
*M* *_r_* = 446.81
Monoclinic, 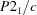

*a* = 10.9124 (3) Å
*b* = 20.3261 (6) Å
*c* = 7.9722 (2) Å
β = 105.965 (2)°
*V* = 1700.08 (8) Å^3^
*Z* = 4
Mo *K*α radiation
μ = 1.54 mm^−1^
*T* = 200 K
0.47 × 0.33 × 0.20 mm

### Data collection  

Stoe IPDS-2 diffractometer
Absorption correction: numerical (*X-SHAPE* and *X-RED 32*; Stoe, 2008 ▸) *T*_min_ = 0.526, *T*_max_ = 0.672
25370 measured reflections
3597 independent reflections
3259 reflections with *I* > 2σ(*I*)
*R*_int_ = 0.063

### Refinement  

*R*[*F* ^2^ > 2σ(*F* ^2^)] = 0.025
*wR*(*F* ^2^) = 0.058
*S* = 1.12
3597 reflections
245 parameters
H-atom parameters constrained
Δρ_max_ = 0.39 e Å^−3^
Δρ_min_ = −0.44 e Å^−3^

### 

Data collection: *X-AREA* (Stoe, 2008 ▸); cell refinement: *X-AREA*; data reduction: *X-AREA*; program(s) used to solve structure: *SHELXS97* (Sheldrick, 2008 ▸); program(s) used to refine structure: *SHELXL2013* (Sheldrick, 2015 ▸); molecular graphics: *XP* in *SHELXTL* (Sheldrick, 2008 ▸) and *DIAMOND* (Brandenburg, 1999 ▸); software used to prepare material for publication: *publCIF* (Westrip, 2010 ▸).

## Supplementary Material

Crystal structure: contains datablock(s) I, global. DOI: 10.1107/S2056989015008890/wm5156sup1.cif

**Fig. 1.**
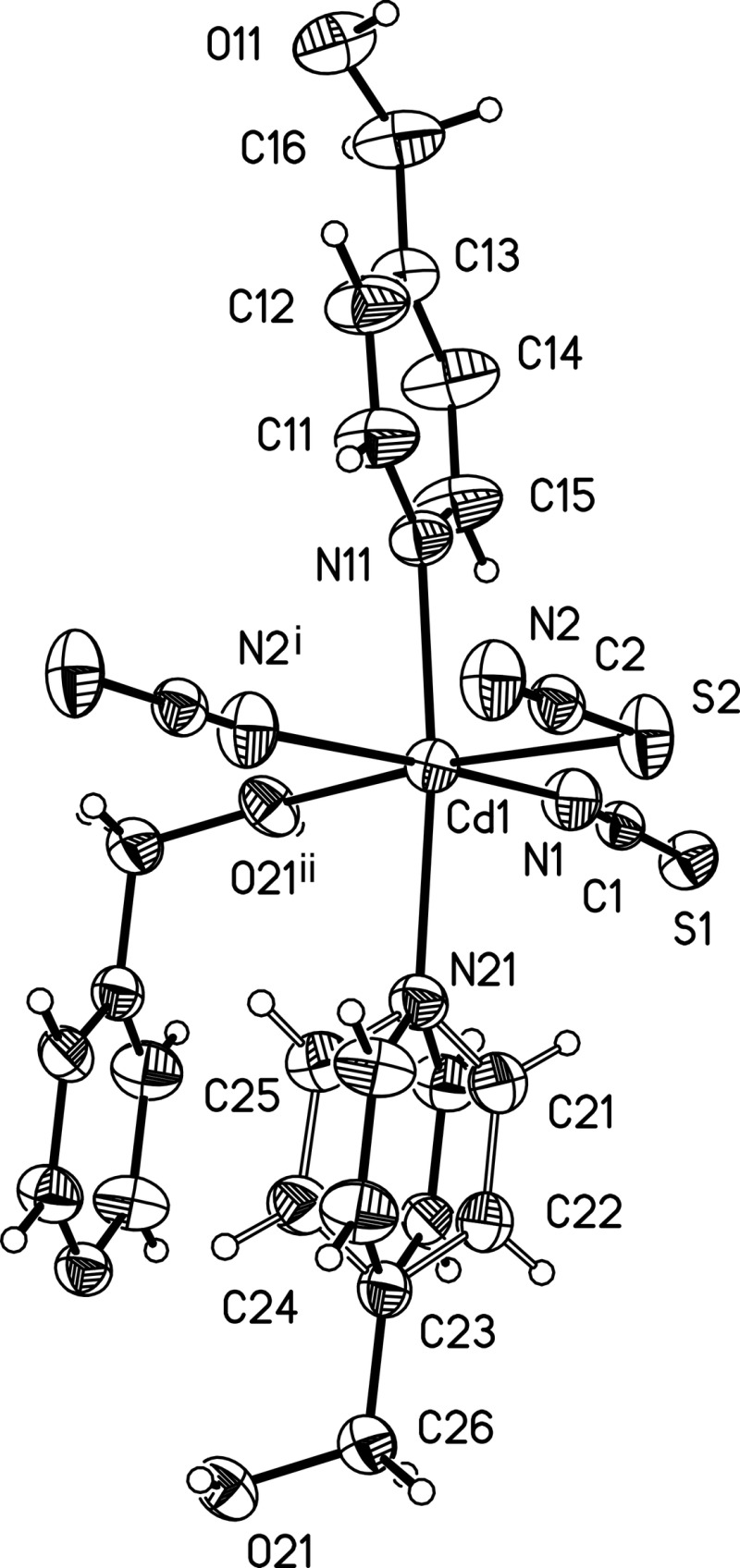
Part of the crystal structure of the title compound with labelling and displacement ellipsoids drawn at the 50% probability level. [Symmetry codes: (i) -x + 1, -y + 1, -z + 1; (ii) -x + 2, -y + 1, -z + 1.]

Structure factors: contains datablock(s) I. DOI: 10.1107/S2056989015008890/wm5156Isup2.hkl

x y z x y z . DOI: 10.1107/S2056989015008890/wm5156fig1.tif
Part of the crystal structure of the title compound with labelling and displacement ellipsoids drawn at the 50% probability level. [Symmetry codes: (i) −*x* + 1, −*y* + 1, −*z* + 1; (ii) −*x* + 2, −*y* + 1, −*z* + 1.]

**Fig. 2.**
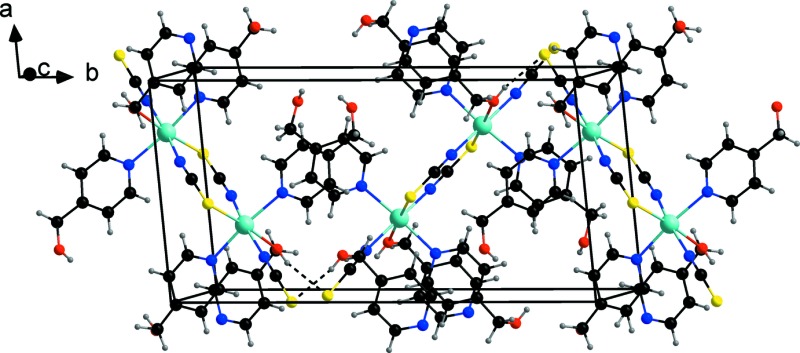
Crystal structure of the title compound in a view approximately along [001]. Intermolecular O—H···S hydrogen bonding is shown as dashed lines; the disordered pyridine rings are omitted for clarity.

. DOI: 10.1107/S2056989015008890/wm5156fig2.tif
Crystal structure of the title compound in a view approximately along [001]. Inter­molecular O—H⋯S hydrogen bonding is shown as dashed lines; the disordered pyridine rings are omitted for clarity.

CCDC reference: 1063786

Additional supporting information:  crystallographic information; 3D view; checkCIF report

## Figures and Tables

**Table 1:** Hydrogen-bond geometry (, )

*D*H*A*	*D*H	H*A*	*D* *A*	*D*H*A*
O11H11*O*S1^i^	0.84	2.49	3.330(2)	174
O21H21*O*S1^ii^	0.84	2.42	3.2410(18)	164

Symmetry codes: (i) 
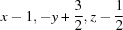
; (ii) 
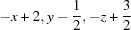
.

## supplementary crystallographic information

### S1. Synthesis and crystallization

CdSO_4_·3/8H_2_O was purchased from Merck and 4-(hy­droxy­methyl)­pyridine and Ba(NCS)_2_·3H_2_O were purchased from Alfa Aesar. Cd(NCS)_2_ was synthesized by stirring 17.5 g (57.0 mmol) Ba(NCS)_2_·3H_2_O and 14.6 g (57.0 mmol) CdSO_4_·3/8H_2_O in 300 ml water at RT for three hours. The white residue of BaSO_4_ was filtered off and dried at 353 K. The homogeneity of the product was checked by X-ray powder diffraction and elemental analysis. The title compound was prepared by the reaction of 34.3 mg (0.15 mmol) Cd(NCS)_2_ and 32.7 mg (0.30 mmol) 4-(hy­droxy­methyl)­pyridine in 1.5 ml methanol at RT. After one week suitable crystals of the title compound were obtained.

### S2. Refinement

The carbon-bound hydrogen atoms were positioned with idealized geometry and were refined with *U*_iso_(H) = 1.2*U*_eq_(C) using a riding model with C—H = 0.95 Å for aromatic and C—H = 0.99 Å for methyl­ene H atoms. The oxygen-bound hydrogen atoms were located in a difference map. For the non-coordinating hydroxyl group the H atom was positioned with idealized geometry allowed to rotate but not to tip, and for the coordinating hydroxyl group its bond length was set to an ideal value of 0.84 Å. Finally, these H atoms were refined with *U*_iso_(H) = 1.5*U*_eq_(O) using a riding model. The pyridine ring of one of the 4-(hy­droxy­methyl)­pyridine ligands is disordered and was refined using a split model in two orientations with an occupancy ratio of 0.46:0.54.

### Crystal data

**Table:**

[Cd(NCS)_2_(C_6_H_7_NO)_2_]	*F*(000) = 888
*M**_r_* = 446.81	*D*_x_ = 1.746 Mg m^−^^3^
Monoclinic, *P*2_1_/*c*	Mo *K*α radiation, λ = 0.71073 Å
*a* = 10.9124 (3) Å	θ = 1.9–26.7°
*b* = 20.3261 (6) Å	µ = 1.54 mm^−^^1^
*c* = 7.9722 (2) Å	*T* = 200 K
β = 105.965 (2)°	Block, colorless
*V* = 1700.08 (8) Å^3^	0.47 × 0.33 × 0.20 mm
*Z* = 4	

### Data collection

**Table:**

Stoe IPDS-2 diffractometer	3259 reflections with *I* > 2σ(*I*)
ω scans	*R*_int_ = 0.063
Absorption correction: numerical (*X-SHAPE* and *X-RED 32*; Stoe, 2008)	θ_max_ = 26.7°, θ_min_ = 1.9°
*T*_min_ = 0.526, *T*_max_ = 0.672	*h* = −13→13
25370 measured reflections	*k* = −25→25
3597 independent reflections	*l* = −10→10

### Refinement

**Table:**

Refinement on *F*^2^	0 restraints
Least-squares matrix: full	Hydrogen site location: mixed
*R*[*F*^2^ > 2σ(*F*^2^)] = 0.025	H-atom parameters constrained
*wR*(*F*^2^) = 0.058	*w* = 1/[σ^2^(*F*_o_^2^) + (0.0217*P*)^2^ + 1.0598*P*] where *P* = (*F*_o_^2^ + 2*F*_c_^2^)/3
*S* = 1.12	(Δ/σ)_max_ = 0.003
3597 reflections	Δρ_max_ = 0.39 e Å^−^^3^
245 parameters	Δρ_min_ = −0.44 e Å^−^^3^

### Special details

**Table:**

Geometry. All esds (except the esd in the dihedral angle between two l.s. planes) are estimated using the full covariance matrix. The cell esds are taken into account individually in the estimation of esds in distances, angles and torsion angles; correlations between esds in cell parameters are only used when they are defined by crystal symmetry. An approximate (isotropic) treatment of cell esds is used for estimating esds involving l.s. planes.

### Fractional atomic coordinates and isotropic or equivalent isotropic displacement parameters (Å^2^)

**Table:**

	*x*	*y*	*z*	*U*_iso_*/*U*_eq_	Occ. (<1)
Cd1	0.70099 (2)	0.59480 (2)	0.62587 (2)	0.02720 (6)	
N1	0.8330 (2)	0.65716 (10)	0.8375 (3)	0.0371 (5)	
C1	0.9127 (2)	0.68944 (11)	0.9253 (3)	0.0274 (5)	
S1	1.02442 (6)	0.73463 (3)	1.05226 (9)	0.03659 (15)	
N2	0.4160 (2)	0.45729 (11)	0.6240 (3)	0.0390 (5)	
C2	0.4891 (2)	0.49106 (11)	0.7172 (3)	0.0291 (5)	
S2	0.59224 (7)	0.53855 (3)	0.85273 (8)	0.04034 (16)	
N11	0.5601 (2)	0.68370 (10)	0.5577 (3)	0.0338 (5)	
C11	0.4387 (3)	0.67718 (14)	0.4676 (4)	0.0477 (7)	
H11	0.4082	0.6342	0.4318	0.057*	
C12	0.3544 (3)	0.72883 (15)	0.4229 (4)	0.0515 (8)	
H12	0.2685	0.7212	0.3582	0.062*	
C13	0.3958 (3)	0.79188 (13)	0.4729 (4)	0.0396 (6)	
C14	0.5211 (3)	0.79861 (14)	0.5691 (5)	0.0587 (9)	
H14	0.5536	0.8409	0.6090	0.070*	
C15	0.5992 (3)	0.74464 (13)	0.6074 (5)	0.0521 (8)	
H15	0.6855	0.7509	0.6726	0.062*	
C16	0.3100 (3)	0.85176 (15)	0.4315 (5)	0.0578 (9)	
H16A	0.2975	0.8690	0.5418	0.069*	
H16B	0.3538	0.8863	0.3821	0.069*	
O11	0.1919 (2)	0.84038 (11)	0.3164 (3)	0.0560 (6)	
H11O	0.1440	0.8225	0.3695	0.084*	
N21	0.8576 (2)	0.51330 (10)	0.6674 (3)	0.0331 (5)	
C21	0.9847 (8)	0.5311 (4)	0.7358 (11)	0.0369 (17)	0.46
H21	1.0044	0.5753	0.7720	0.044*	0.46
C22	1.0837 (8)	0.4867 (4)	0.7529 (10)	0.0371 (17)	0.46
H22	1.1695	0.5001	0.8019	0.045*	0.46
C23	1.0562 (2)	0.42284 (12)	0.6982 (3)	0.0315 (5)	
C24	0.9315 (9)	0.4061 (4)	0.6329 (12)	0.0421 (19)	0.46
H24	0.9090	0.3620	0.5981	0.050*	0.46
C25	0.8377 (10)	0.4528 (5)	0.6170 (13)	0.040 (2)	0.46
H25	0.7521	0.4396	0.5653	0.048*	0.46
C21'	0.9611 (8)	0.5162 (4)	0.7970 (9)	0.0456 (18)	0.54
H21'	0.9666	0.5494	0.8825	0.055*	0.54
C22'	1.0627 (8)	0.4740 (4)	0.8169 (9)	0.0435 (17)	0.54
H22'	1.1371	0.4797	0.9113	0.052*	0.54
C24'	0.9438 (7)	0.4180 (3)	0.5626 (9)	0.0372 (15)	0.54
H24'	0.9342	0.3840	0.4781	0.045*	0.54
C25'	0.8466 (9)	0.4627 (4)	0.5515 (10)	0.0365 (17)	0.54
H25'	0.7697	0.4581	0.4606	0.044*	0.54
C26	1.1645 (3)	0.37527 (14)	0.7161 (4)	0.0394 (6)	
H26A	1.2445	0.3965	0.7837	0.047*	
H26B	1.1497	0.3364	0.7828	0.047*	
O21	1.17960 (17)	0.35385 (8)	0.5527 (2)	0.0352 (4)	
H21O	1.1185	0.3283	0.5100	0.053*	

### Atomic displacement parameters (Å^2^)

**Table:**

	*U*^11^	*U*^22^	*U*^33^	*U*^12^	*U*^13^	*U*^23^
Cd1	0.02612 (10)	0.02547 (9)	0.02941 (10)	−0.00284 (6)	0.00661 (7)	−0.00213 (6)
N1	0.0362 (12)	0.0363 (11)	0.0353 (11)	−0.0040 (10)	0.0041 (10)	−0.0081 (9)
C1	0.0310 (12)	0.0257 (10)	0.0268 (11)	0.0038 (9)	0.0101 (10)	0.0012 (9)
S1	0.0352 (3)	0.0342 (3)	0.0357 (3)	−0.0051 (3)	0.0018 (3)	−0.0052 (3)
N2	0.0413 (13)	0.0408 (11)	0.0337 (11)	−0.0132 (10)	0.0081 (10)	−0.0055 (9)
C2	0.0319 (13)	0.0285 (11)	0.0298 (12)	0.0006 (10)	0.0135 (10)	0.0028 (9)
S2	0.0436 (4)	0.0491 (4)	0.0293 (3)	−0.0211 (3)	0.0117 (3)	−0.0072 (3)
N11	0.0285 (11)	0.0324 (10)	0.0381 (11)	−0.0011 (8)	0.0052 (9)	−0.0008 (9)
C11	0.0339 (15)	0.0357 (14)	0.0641 (19)	0.0000 (11)	−0.0025 (13)	−0.0130 (13)
C12	0.0317 (15)	0.0474 (16)	0.064 (2)	0.0022 (12)	−0.0054 (14)	−0.0131 (14)
C13	0.0342 (14)	0.0353 (13)	0.0474 (15)	0.0020 (11)	0.0083 (12)	0.0023 (11)
C14	0.0390 (17)	0.0307 (13)	0.093 (3)	−0.0030 (12)	−0.0039 (17)	−0.0012 (15)
C15	0.0320 (15)	0.0327 (13)	0.080 (2)	−0.0039 (11)	−0.0046 (15)	−0.0001 (14)
C16	0.0403 (17)	0.0426 (16)	0.082 (2)	0.0062 (13)	0.0033 (16)	0.0040 (16)
O11	0.0403 (12)	0.0575 (13)	0.0648 (14)	0.0061 (10)	0.0053 (10)	0.0117 (11)
N21	0.0369 (12)	0.0297 (10)	0.0348 (11)	0.0041 (9)	0.0132 (9)	0.0021 (8)
C21	0.032 (4)	0.033 (3)	0.043 (5)	−0.002 (3)	0.005 (3)	−0.012 (3)
C22	0.031 (3)	0.048 (4)	0.032 (5)	0.006 (3)	0.008 (3)	−0.009 (3)
C23	0.0361 (13)	0.0318 (11)	0.0301 (12)	0.0034 (10)	0.0150 (10)	0.0053 (9)
C24	0.044 (4)	0.026 (3)	0.057 (6)	−0.002 (3)	0.014 (4)	−0.003 (4)
C25	0.029 (4)	0.029 (4)	0.063 (7)	−0.004 (3)	0.016 (5)	0.000 (4)
C21'	0.051 (5)	0.050 (4)	0.032 (4)	0.014 (3)	0.005 (3)	−0.009 (3)
C22'	0.044 (4)	0.052 (4)	0.028 (4)	0.012 (3)	0.001 (3)	−0.009 (3)
C24'	0.041 (3)	0.031 (3)	0.038 (4)	0.004 (2)	0.009 (3)	−0.005 (3)
C25'	0.035 (4)	0.031 (3)	0.041 (4)	−0.001 (3)	0.005 (3)	−0.003 (3)
C26	0.0435 (15)	0.0432 (14)	0.0353 (14)	0.0099 (12)	0.0170 (12)	0.0054 (11)
O21	0.0388 (10)	0.0305 (8)	0.0427 (10)	−0.0039 (7)	0.0217 (8)	−0.0027 (7)

### Geometric parameters (Å, º)

**Table:**

Cd1—N1	2.279 (2)	N21—C25	1.294 (11)
Cd1—N2^i^	2.306 (2)	N21—C21'	1.305 (8)
Cd1—N11	2.337 (2)	N21—C25'	1.366 (9)
Cd1—N21	2.337 (2)	N21—C21	1.392 (9)
Cd1—O21^ii^	2.4153 (17)	C21—C22	1.385 (12)
Cd1—S2	2.6771 (7)	C21—H21	0.9500
N1—C1	1.158 (3)	C22—C23	1.376 (9)
C1—S1	1.636 (2)	C22—H22	0.9500
N2—C2	1.155 (3)	C23—C24	1.360 (9)
N2—Cd1^i^	2.306 (2)	C23—C22'	1.395 (8)
C2—S2	1.643 (2)	C23—C24'	1.398 (8)
N11—C11	1.329 (3)	C23—C26	1.503 (4)
N11—C15	1.334 (3)	C24—C25	1.376 (14)
C11—C12	1.376 (4)	C24—H24	0.9500
C11—H11	0.9500	C25—H25	0.9500
C12—C13	1.381 (4)	C21'—C22'	1.376 (11)
C12—H12	0.9500	C21'—H21'	0.9500
C13—C14	1.379 (4)	C22'—H22'	0.9500
C13—C16	1.516 (4)	C24'—C25'	1.381 (12)
C14—C15	1.371 (4)	C24'—H24'	0.9500
C14—H14	0.9500	C25'—H25'	0.9500
C15—H15	0.9500	C26—O21	1.426 (3)
C16—O11	1.380 (4)	C26—H26A	0.9900
C16—H16A	0.9900	C26—H26B	0.9900
C16—H16B	0.9900	O21—Cd1^ii^	2.4152 (17)
O11—H11O	0.8400	O21—H21O	0.8400
			
N1—Cd1—N2^i^	169.07 (8)	C21'—N21—C25'	117.8 (5)
N1—Cd1—N11	89.06 (7)	C25—N21—C21	115.7 (6)
N2^i^—Cd1—N11	88.96 (8)	C25—N21—Cd1	125.2 (5)
N1—Cd1—N21	90.03 (8)	C21'—N21—Cd1	121.3 (4)
N2^i^—Cd1—N21	90.36 (8)	C25'—N21—Cd1	120.8 (4)
N11—Cd1—N21	171.60 (7)	C21—N21—Cd1	118.8 (4)
N1—Cd1—O21^ii^	82.07 (7)	C22—C21—N21	122.3 (7)
N2^i^—Cd1—O21^ii^	87.11 (7)	C22—C21—H21	118.8
N11—Cd1—O21^ii^	87.47 (7)	N21—C21—H21	118.8
N21—Cd1—O21^ii^	84.13 (7)	C23—C22—C21	119.2 (7)
N1—Cd1—S2	92.58 (6)	C23—C22—H22	120.4
N2^i^—Cd1—S2	98.31 (6)	C21—C22—H22	120.4
N11—Cd1—S2	95.85 (6)	C24—C23—C22	117.8 (5)
N21—Cd1—S2	92.54 (5)	C22'—C23—C24'	116.6 (5)
O21^ii^—Cd1—S2	173.68 (5)	C24—C23—C26	123.6 (4)
C1—N1—Cd1	168.3 (2)	C22—C23—C26	118.7 (4)
N1—C1—S1	179.0 (2)	C22'—C23—C26	121.5 (4)
C2—N2—Cd1^i^	161.6 (2)	C24'—C23—C26	121.9 (4)
N2—C2—S2	179.0 (2)	C23—C24—C25	120.2 (8)
C2—S2—Cd1	99.07 (9)	C23—C24—H24	119.9
C11—N11—C15	116.3 (2)	C25—C24—H24	119.9
C11—N11—Cd1	122.87 (17)	N21—C25—C24	124.8 (9)
C15—N11—Cd1	120.82 (17)	N21—C25—H25	117.6
N11—C11—C12	124.0 (3)	C24—C25—H25	117.6
N11—C11—H11	118.0	N21—C21'—C22'	123.9 (7)
C12—C11—H11	118.0	N21—C21'—H21'	118.1
C11—C12—C13	119.4 (3)	C22'—C21'—H21'	118.1
C11—C12—H12	120.3	C21'—C22'—C23	119.8 (6)
C13—C12—H12	120.3	C21'—C22'—H22'	120.1
C14—C13—C12	116.6 (3)	C23—C22'—H22'	120.1
C14—C13—C16	120.0 (3)	C25'—C24'—C23	119.9 (6)
C12—C13—C16	123.3 (3)	C25'—C24'—H24'	120.0
C15—C14—C13	120.4 (3)	C23—C24'—H24'	120.0
C15—C14—H14	119.8	N21—C25'—C24'	121.9 (7)
C13—C14—H14	119.8	N21—C25'—H25'	119.0
N11—C15—C14	123.2 (3)	C24'—C25'—H25'	119.0
N11—C15—H15	118.4	O21—C26—C23	113.3 (2)
C14—C15—H15	118.4	O21—C26—H26A	108.9
O11—C16—C13	114.7 (3)	C23—C26—H26A	108.9
O11—C16—H16A	108.6	O21—C26—H26B	108.9
C13—C16—H16A	108.6	C23—C26—H26B	108.9
O11—C16—H16B	108.6	H26A—C26—H26B	107.7
C13—C16—H16B	108.6	C26—O21—Cd1^ii^	128.55 (16)
H16A—C16—H16B	107.6	C26—O21—H21O	106.3
C16—O11—H11O	109.5	Cd1^ii^—O21—H21O	121.7

Symmetry codes: (i) −*x*+1, −*y*+1, −*z*+1; (ii) −*x*+2, −*y*+1, −*z*+1.

### Hydrogen-bond geometry (Å, º)

**Table:**

*D*—H···*A*	*D*—H	H···*A*	*D*···*A*	*D*—H···*A*
O11—H11*O*···S1^iii^	0.84	2.49	3.330 (2)	174
O21—H21*O*···S1^iv^	0.84	2.42	3.2410 (18)	164

Symmetry codes: (iii) *x*−1, −*y*+3/2, *z*−1/2; (iv) −*x*+2, *y*−1/2, −*z*+3/2.
