# Supplementary figures and images for: Crystal structure of poly[[μ-4-(hy­droxy­meth­yl)pyridine-κ2 N:O][4-(hy­droxy­meth­yl)pyridine-κN](μ-thio­cyanato-κ2 N:S)(thio­cyanato-κN)cadmium]
Source: Acta Crystallogr E Crystallogr Commun. 2015 May 13;71(Pt 6):m129–30. doi: 10.1107/S2056989015008890 (PMC4459314; doi:10.1107/S2056989015008890)

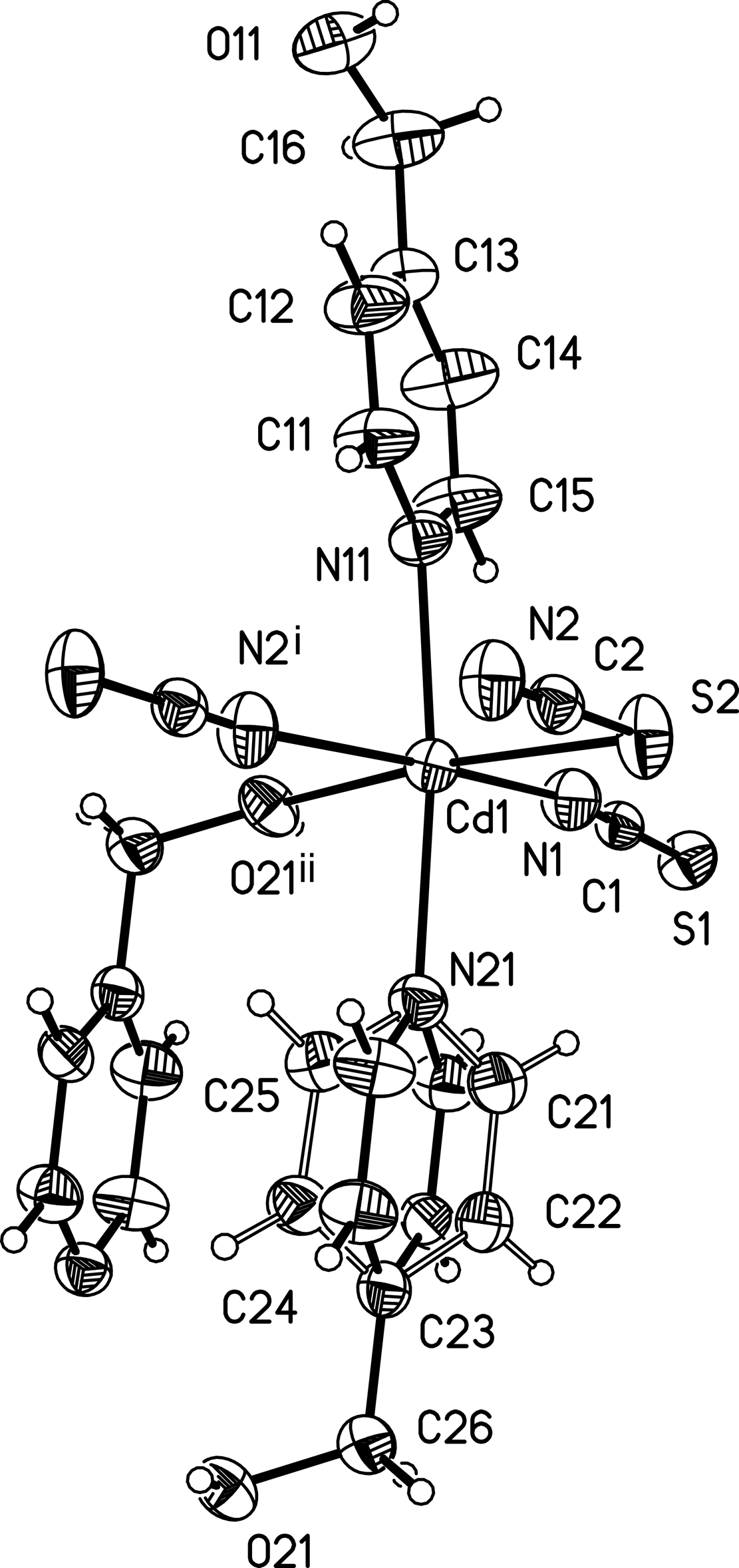

Supplement: Supplementary file 3 [file e-71-0m129-fig1.tif]

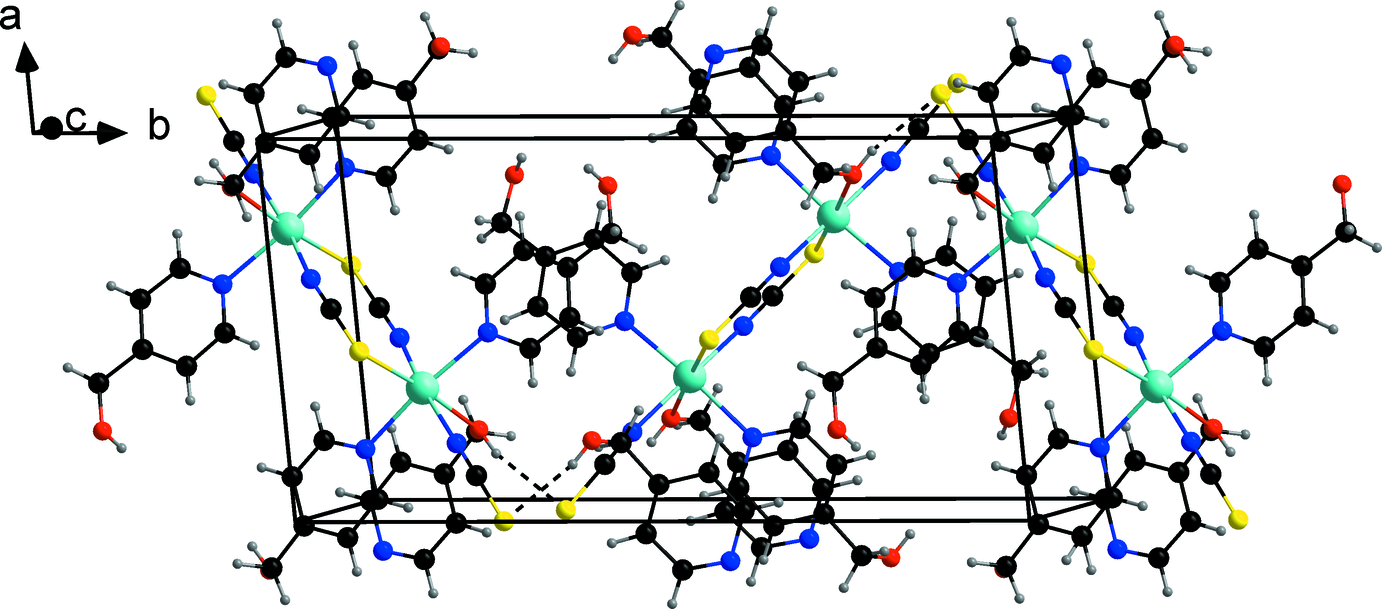

Supplement: Supplementary file 4 [file e-71-0m129-fig2.tif]
